# A Case of Infantile Cardiac Rhabdomyoma Complicated by Tuberous Sclerosis

**DOI:** 10.4021/cr104e

**Published:** 2010-11-20

**Authors:** Takehiro Serikawa, Yasuhiro Takahashi, Akira Kikuchi, Koichi Takakuwa, Tohei Usuda, Satoshi Hasegawa, Kenichi Tanaka

**Affiliations:** a Department of Obstetrics and Gynecology, Niigata University Medical and Dental Hospital, Niigata, Japan; b Department of Pediatrics, Niigata University Medical and Dental Hospital, Niigata, Japan

**Keywords:** Fetal cardiac tumor, Rhabdomyoma, Prenatal diagnosis, Tuberous sclerosis

## Abstract

We experienced a case with fetal cardiac tumor, which was diagnosed by prenatal ultrasonographic examination, and the diagnosis was confirmed after birth. A pregnancy woman of the 26th week of gestation was referred to our hospital for close examinations of fetal cardiac tumor. Ultrasonographic examinations revealed single homogeneous tumor with the diameter of 14 mm intracardiac space. The tumor was considered to emerge from the ventricular septum and to be occupied in left ventricle. Other cardiac abnormalities were not detected. The fetus was diagnosed to be complicated with the intracardiac tumor, and with the possible rhabdomyoma of heart. The serial ultrasonographic examinations revealed that the fetal cardiac function was normal. The size of the tumor gradually increased, although the fetal cardiac function revealed within normal range. The patient delivered a female infant weighing 2716g with the Apgar score of 9 and 10 at one and 5 minutes after delivery. The infant was confirmed to have cardiac tumors after examination by pediatric cardiologist, and the cardiac function of the infant was diagnosed as normal condition. The computed tomography of the head revealed the intracranial multiple calcification lesions, which indicated the symptoms of tuberous sclerosis.

## Introduction

Fetal or neonatal cardiac rhabdomyoma is a benign, smooth muscle tumor of the myocardium consisting of immature myocytes. It is well recognized that about 50 to 60% of cardiac rhabdomyomas are associated with tuberous sclerosis, which is inherited via the autosomal-dominant Mendelian system. The remaining 40 to 50% of cases of neonatal cardiac rhabdomyoma emerge in patients without tuberous sclerosis. We cared for a case of fetal cardiac tumor which was diagnosed in the prenatal period by means of ultrasonographic examination. The affected infant was diagnosed with tuberous sclerosis during the neonatal period, although the family history revealed no cases of tuberous sclerosis. In this report, we present the clinical course of this case and discuss the importance of prenatal diagnosis of fetal cardiac tumors.

## Case Report

**Figure 1:**
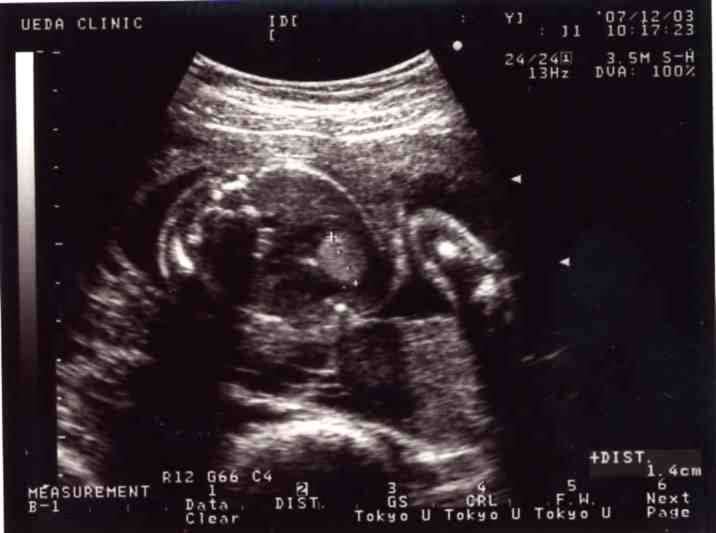
Four-chamber view of the fetal heart. A large homogeneous tumor occupies the left ventricle. A tumor is not contiguous with the ventricular septum.

A 27-year-old Japanese woman in the 26th week of gestation (gravida 1, para 0) was referred to the Obstetric Outpatient Clinic at Niigata University Medical and Dental Hospital for close examination of a fetal cardiac tumor. The patient had no complications, no genetic diseases, and had conceived spontaneously. She had undergone prenatal management and care at the hospital near her residence. Ultrasonographic examinations performed during the 25th week of gestation by the referring hospital demonstrated a fetal cardiac tumor. Ultrasonographic examinations performed at the first visit revealed a single, homogeneous tumor 14 mm in diameter in the intracardic space (Fig. 1). The tumor was believed to emerge from the ventricular septum and to occupy the left ventricle. A significant pericardial effusion was noted. The ventricular and atrial rates were 140 beats per minute and no atrioventricular block was observed. Other cardiac abnormalities were not detected, and the velocity of blood flow in the aorta and pulmonary arteries was within the normal range. The fetus was diagnosed with an intracardic tumor, possibly cardiac rhabdomyoma. Pedigree analyses revealed no family history of tuberous sclerosis.

**Figure 2:**
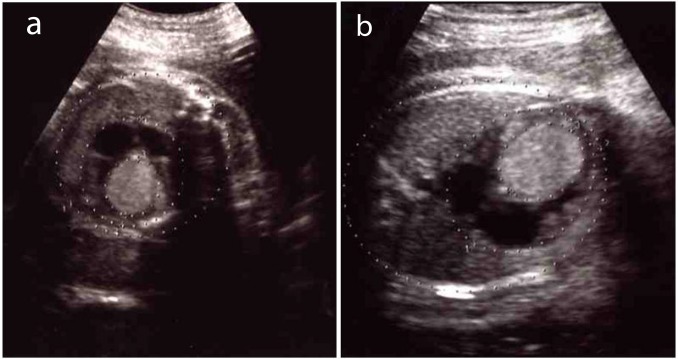
A change in the size of the tumor. (a) Tumor diameter was 22 mm at the 32nd week of gestation. (b) It gradually increased to 24 mm at the 35th week of gestation.

The patient was managed in the outpatient clinic at our hospital. Serial ultrasonographic examinations were performed and fetal cardiac function was investigated. The size of the fetal cardiac tumor gradually increased (Fig. 2a, 2b), although the fetal cardiac function was revealed to be within the normal range. The patient was hospitalized with the onset of spontaneous labor pain in the 39th week of gestation, and a female infant weighing 2716 g was delivered with Apgar scores of nine and ten at one and five minutes after delivery, respectively. The patient’s puerperal course was uneventful, and she was discharged from our hospital at the sixth day postpartum.

**Figure 3:**
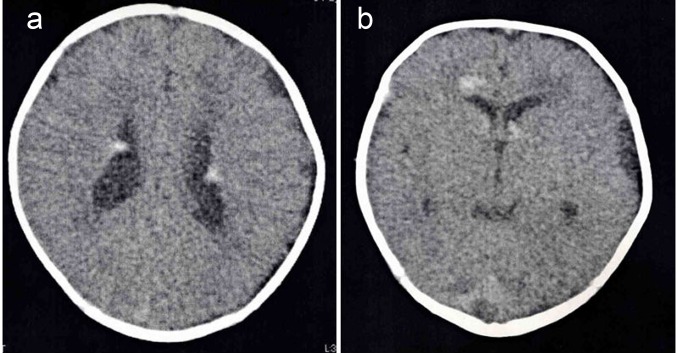
CT of the head. There were multiple sclerosed regions at the wall of lateral ventricle (a) and foramen of Monro (b).

After delivery, examination by a pediatric cardiologist confirmed that the infant had a cardiac tumor and the cardiac function of the infant was diagnosed as normal. There were multiple tumors; the largest tumor with a diameter of 26 mm was in the left ventricle and tumors with diameters of 5 to 15 mm were in the right ventricle, on the aortic and tricuspid valves, and at the apex. There was no evidence of outflow tract obstruction or hemodynamic compromise. The infant’s postpartum weight gain was observed to be normal. Computed tomography (CT) of the head revealed multiple calcified intracranial lesions (Fig. 3a, 3b), which are symptomatic of tuberous sclerosis. Therefore, the infant was diagnosed with tuberous sclerosis. No surgical treatment was performed as there were no indications. The infant was discharged and has been managed at the outpatient pediatric clinic at our hospital.

## Discussion

Fetal cardiac rhabdomyoma is a rare condition, but it is the most common fetal cardiac tumor. More than 60% of antenatally detected cardiac tumors are rhabdomyomas and these are often associated with tuberous sclerosis [1]. The definite diagnosis in this case was cardiac rhabdomyoma associated with tuberous sclerosis, following close examination after birth. Although tuberous sclerosis is generally reported to be an autosomal-dominant, multisystem disorder with variable expressivity [2], it seemed that this case was sporadic as there was no family history.

In the absence of a positive family history, prenatal diagnosis of tuberous sclerosis depends on the detection of cardiac rhabdomyomas. Cardiac rhabdomyomas with multiple tumors are associated with the tuberous sclerosis in 50-80% of cases [1]. The appearance of rhabdomyomas by ultrasound is characterized by round, homogenous, hyperechogenic masses in the ventricles that sometimes appear as multiple foci in the ventricles and septal walls [3]. Although prenatal diagnosis of the complicating tuberous sclerosis was difficult in this case, it was possible to diagnose the tumors as rhabdomyomas because there were multiple homogenous tumors, including a large tumor in the left ventricle, one on the aortic valve, one at the cardiac apex, two in the right ventricle, and one on the tricuspid valve. We performed serial, close ultrasonographic examinations for this case because occasionally this kind of cardiac tumor shrinks naturally [4]. Although the main, large tumor did not decrease in volume during the prenatal course, the fetus did not have any arrhythmias or heart failure so we could await fetal maturity before delivery. After birth, CT of the head revealed multiple, calcified intracranial lesions (Fig. 3) indicative of tuberous sclerosis. Therefore, the infant was diagnosed with tuberous sclerosis.

We experienced a case of fetal cardiac tumor diagnosed in the prenatal period by means of ultrasonographic examination. The affected infant was diagnosed with tuberous sclerosis despite a negative family history. Infants with tuberous sclerosis are at risk of epilepsy or mental retardation during the infantile period; and the early diagnosis and initiation of management of the disease is considered to be crucial. Thus, the obstetrician should always remain vigilant to decide whether there is the possibility of tuberous sclerosis in cases of fetal cardiac tumors.
